# Impact of seasons and heat waves on the incidence of *Staphylococcus aureus* and *Escherichia coli* bacteremia – A prospective multicenter study using biometeorological data

**DOI:** 10.1371/journal.pone.0352186

**Published:** 2026-07-14

**Authors:** Philipp Mathé, Kathrin Graw, Andreas Matzarakis, Máté Elod Maros, David Tobys, Britta Kohlmorgen, Nadja Käding, Silke Peter, Harald Seifert, Petra Gastmeier, Jan Rupp, Siri Göpel, Can Imirzalioglu, Daniel Hornuss, Gabriele Peyerl-Hoffmann, Insa Joost, Evelina Tacconelli, Winfried V. Kern, Martin Wolkewitz, Siegbert Rieg, DZIF R-Net Study Group

**Affiliations:** 1 DZIF German Centre for Infection Research, Braunschweig, Germany; 2 Division of Infectious Diseases, Department of Medicine II, Faculty of Medicine, Medical Center – University of Freiburg, University of Freiburg, Freiburg, Germany; 3 Research Centre Human Biometeorology, German Meteorological Service, Freiburg, Germany; 4 Chair of Environmental Meteorology, Faculty of Natural Resources and Environment, University of Freiburg, Freiburg, Germany; 5 Democritus University of Thrace, Komotini, Greece; 6 Department of Biomedical Informatics at the Mannheim Institute for intelligent Systems in Medicine (MIISM), Medical Faculty Mannheim, Heidelberg University, Mannheim, Germany; 7 Faculty of Medicine and University Hospital Cologne, Institute for Medical Microbiology, Immunology and Hygiene, University of Cologne, Cologne, Germany; 8 Institute for Hygiene and Environmental Medicine, National Reference Centre for the Surveillance of Nosocomial Infections, Charité-University Hospital, Berlin, Germany; 9 Infectious Diseases Clinic, University of Lübeck and University Hospital Schleswig-Holstein, Lübeck, Germany; 10 Institute of Medical Microbiology and Hygiene, University Hospital Tübingen, Tübingen, Germany; 11 Faculty of Medicine and University Hospital Cologne, Institute of Translational Research, Cologne Excellence Cluster on Cellular Stress Responses in Aging-Associated Diseases (CECAD), University of Cologne, Cologne, Germany; 12 Institute of Medical Microbiology, University of Lübeck and University Hospital Schleswig-Holstein, Lübeck, Germany; 13 Department of Internal Medicine I, University Hospital Tübingen, Tübingen, Germany; 14 Institute of Medical Microbiology, Justus-Liebig-University Giessen, Gießen, Germany; 15 Institute of Medical Microbiology and Hospital Hygiene, Heinrich-Heine-University Düsseldorf, Düsseldorf, Germany; 16 Division of Infectious Diseases, Department of Diagnostic and Public Health, University of Verona, Policlinico GB Rossi, Verona, Italy; 17 Faculty of Medicine and Medical Center, Institute of Medical Biometry and Statistics, University of Freiburg, Freiburg, Germany; Tel Aviv University School of Medicine, ISRAEL

## Abstract

**Objectives:**

Climate change and global warming are major threats for human health and impact the burden of infectious diseases. We investigated the effect of heat stress days (max. perceived temperature ≥32°C) on the incidence of *Staphylococcus aureus* bacteremia (SAB) and *Escherichia coli* bacteremia (ECB).

**Methods:**

We performed a post-hoc analysis of a prospective multicenter cohort study with inclusion of all reported SAB and ECB episodes at six tertiary care centers in Germany from 01/2017–12/2019. The effect of the number of heat stress days on the incidence of bacteremia episodes was modelled by a negative binomial regression model with and without underlying seasonal trend.

**Results:**

In the prospective multicenter cohort, we included 2870 episodes of SAB and 4421 episodes of ECB. For both entities, we found a significant seasonal variation over the year (ECB peak-to-trough ratio: 1.33, 95% CI: 1.23–1.45, p < 0.001); SAB peak-to-trough ratio: 1.19 (95% CI: 1.07–1.32, p < 0.001), especially in the subgroup of community-acquired ECB (1.47, 95%-CI: 1.32–1.64, p < 0.001). In the model with incorporation of an underlying seasonal trend, we discovered no overall significant association with the number of heat stress days for SAB and ECB. However, in the subgroup of patients with hospital-acquired SAB, we found a significant association after two days of heat (IRR 1.45, 95%-CI: 1.17–1.82, p = 0.001), that remained significant also in a sensitivity analysis focusing on summer days only (IRR 1.50, 95%-CI: 1.18–1.91, p = 0.001). However, after inclusion of an underlying seasonal trend, the incidence of bacteremia cases remained significantly associated with heat stress days only in the subgroup of patients with hospital-acquired SAB.

**Conclusion:**

Apart from seasonal trends, heat days did not seem to influence the incidence of SAB and ECB overall. The observed association of SAB with heat days in the subgroup of hospital-acquired SAB needs confirmation in further studies.

## Introduction

Seasonal influences have been known to be an important determinant of disease since the age of Hippocrates. Several studies in the last years have shown that external factors such as season, temperature or humidity may influence the rate of pneumonia, bloodstream and wound infections and the prevalence of antibiotic-resistant bacteria [1–7]. Significant seasonality has been shown for some pathogens, e.g., *Streptococcus pneumoniae* or *Escherichia coli* [8].

*Staphylococcus aureus* is a major pathogen of community-acquired and healthcare-associated infections including bacteremia [9]. However, data regarding seasonal influences are contradictory for *S. aureus*. Both, increases and decreases in the rate of *S. aureus* colonization and infection have been observed with increasing outdoor temperature [4,8,10,11]. Previous studies reported a higher MRSA colonization rate of human skin by decreasing latitude [12,13]. The geographic proximity to the equator is associated with higher average temperatures, as well as lower seasonal variation, which suggests a link of *S. aureus* infections to increased temperatures and humidity.

For *E. coli,* seasonality has been described with an increase in the number of infections during summer [4,8,14–17]. This increase was also found for hospital-acquired bacteremias in some studies [5,18], while others reported no significant differences [17]. The causes of this increase are incompletely understood, yet some studies discuss that improved growth conditions for Gram-negative bacilli, resulting in higher colonization levels, could play an important role [4,19].

Given the accelerating climate change, it seems prudent to examine heat separately from seasonality. Heat poses additional health risks by exceeding physiological limits and impairing body functions, while seasonality incorporates a broader range of factors, including general meteorological conditions and changes in human behavior [1]. The specific influence of extreme heat episodes remains unclear. Furthermore, there is a need for further investigation regarding the importance of the duration of heat episodes and the time interval between heat exposure and subsequent infection risk.

We set out to answer whether heat waves or consecutive heat stress days (as phases of constant heat stress) in a setting of temperate climate influence the incidence of two major invasive infectious disease entities, i.e., bacteremia due to *S. aureus* (SAB) and *E. coli* (ECB). Moreover, we aimed to elucidate whether there are differential influences on community-acquired or hospital-acquired SAB or ECB.

## Methods

### Patients and setting

We performed a post-hoc analysis of a prospectively evaluated multicenter cohort study including all reported *Staphylococcus aureus* and *Escherichia coli* bacteremia episodes at six tertiary care hospitals in Germany (Berlin, Cologne, Freiburg, Gießen, Lübeck, Tübingen) between January 2017 and December 2019. All blood cultures growing isolates of *S. aureus* or *E. coli* were reported on a daily basis by the microbiological laboratory. Only patients ≥ 18 years were included.

### Ethical considerations

All IRB of the involved study sites approved the R-Net study protocol (Number 302/14 & 302/14_160765). The ethical standards set by the Helsinki Declaration of 1964, as revised in 2013, were followed. The need for consent was waived by the ethics committee.

### Data acquisition of the clinical data

In our clinical dataset, the first positive blood culture for *S. aureus* or *E. coli* defined a case of bacteremia. Subsequently drawn positive blood cultures of the same patient in the following 30 days were subsidized as one case. Furthermore, every patient was further characterized for age, sex, the mode of acquisition (community-acquired vs. hospital-acquired), the kind of ward at onset (regular ward vs. intensive care unit), the presence of polymicrobial bacteremia and bacterial resistance. The data was retrieved via the electronic health record of each patient.

### Definitions used for clinical data

The date of onset of bacteremia was defined as the date of the first positive blood culture for the respective pathogen. A case was considered community-acquired if the onset of the bacteremia was before or <48h after admission to the hospital, other cases were considered hospital-acquired. Polymicrobial infection was defined as blood cultures with growth of more than one relevant pathogen. The following resistance determinants were recorded, for *S. aureus* resistance to methicillin (MRSA), for *E. coli* resistance to third-generation cephalosporins (3GCREC). The nominal number of patient cases included all patients admitted to the hospital wards (pediatric/psychiatric/dermatological wards excluded) aggregated by year.

### Data acquisition of the meteorological data

The meteorological data originated from measurement stations of the German Meteorological Service (DWD) next to the six tertiary university hospitals, slightly out of town or in the suburban area to diminish urban effects like the urban heat island (UHI) on the measurements. The six meteorological measurement stations with their DWD-Stations-IDs in parentheses were:

- Berlin-Tegel (*430*) for university hospital in Berlin,
- Freiburg (*1443*) at the aerodrome for university hospital in Freiburg im Breisgau,
- Gießen/Wettenberg (*1639*) for university hospital in Gießen,
- Köln/Bonn (*2667*) at the airport for university hospital in Köln,
- Lübeck-Blankensee (*3086*) for university hospital in Lübeck and
- Stuttgart-Echterdingen (*4931*) for university hospital in Tübingen.

For every station the perceived temperature was computed with the heat budget model of the DWD based on air temperature, humidity, wind velocity and the long and short-term radiation to predict the severity of the impact of thermal stress on the human body.

### Definitions used for meteorological data

Heat days were defined as days with a maximum perceived temperature ≥ 32 °C. The number of heat stress days (0–3) was assigned to every date by the number of heat days in the three days period before the respective date.

### Statistical analysis

The clinical data were merged with the meteorological data for consecutive three-day periods. We dropped the last week of the year due to an unlikely low number of reported cases, most likely due to an underreporting during Christmas holidays. Every heat day group was assigned to a consecutive three-day period with aggregation of the number of bacteremias, like shown here:


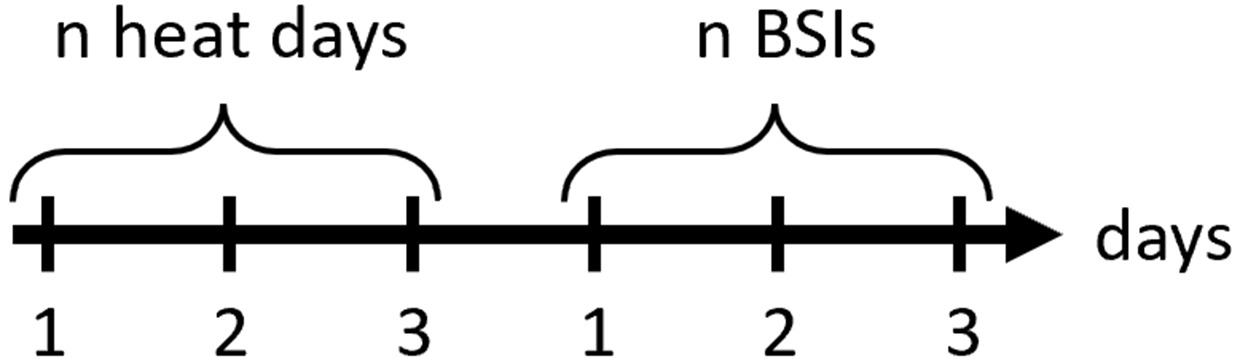


We used a generalized mixed-effects model with a negative binomial distribution to examine the aggregated three-day infection counts and to calculate incidence rate ratios (IRR). The analysis incorporated the following dependent variables: the number of heat days in the lagged time period (0, 1, 2, 3), year (2017, 2018, 2019), and a natural spline of the week with three degrees of freedom as fixed effects, along with the center as a random effect. As sensitivity analysis, we performed another generalized mixed-effects model without inclusion of a natural spline modelling seasonality, with included cases limited to those occurred in summer months (meteorological summer: 1^st^ June – 31^st^ August).

To estimate seasonality we calculated the aggregated case numbers of each pathogen by month corrected for the number of patient cases and calculated the peak-to-trough ratio (ratio of the peak monthly number of events to the lowest monthly number of events) including confidence intervals using Episheet (https://www.drugepi.org/dope/software#Episheet) [20].

All analyses were performed with the R statistics program (v.4.0.2, R Core Team 2024, Vienna Austria; RStudio IDE v. 2022.02.3−492, Boston, MA). Non-normally distributed data were displayed as median and interquartile range (IQR). Categorical variables were reported as proportions. P-values <0.05 were considered significant.

## Results

### Patient characteristics

During the study period, 2870 single episodes of *S. aureus* bacteremia (SAB) and 4421 single episodes of *E. coli* bacteremia (ECB) were detected in all centers, with a mean number of 159.4 cases (range 72−277) for *S. aureus* as well as 245.6 cases (range 146−529) for *E. coli* per year and center (Table 1). In nearly all centers we found an increasing number of detected bacteremias corrected by the number of patient cases between 2017–2019 (SAB average increase +14%, range: −3%- + 38%; ECB average increase +23%, range: −3%- + 52%). Patients were predominantly elderly men (65.5% for SAB; 53.6% for ECB) with community-acquired infection (54.1% for SAB;65.1% for ECB) and treated mainly on internal medicine wards (65.8% for SAB; 71.7% for ECB) as well as on non-ICU wards (70.9% for SAB; 77.2% for ECB). Polymicrobial bacteremia was rare for both pathogens, the proportion of infection by resistant pathogens was low (7.9% MRSA; 15.4% 3GCREC).

**Table 1:** Cohort characteristics.

	*S. aureus*		*E. coli*	
**Variable**	**n (%)**	**Median (IQR)**	**n (%)**	**Median (IQR)**
n total	2870 (100%)		4421 (100%)	
Center A	529/2870 (18.4%)		845/4421 (19.1%)	
Center B	773/2870 (26.9%)		1435/4421 (32.5%)	
Center C	518/2870 (18.0%)		632/4421 (14.3%)	
Center D	388/2870 (13.5%)		487/4421 (11.0%)	
Center E	409/2870 (14.3%)		549/4421 (12.4%)	
Center F	253/2870 (8.8%)		473/4421 (10.7%)	
Year				
2017	943/2870 (32.9%)		1362/4421 (30.8%)	
2018	935/2870 (32.6%)		1470/4421 (33.3%)	
2019	992/2870 (34.6%)		1589/4421 (35.9%)	
**Patient characteristics**				
Age, years		68 (22)		69 (20)
Sex				
*Female*	990/2870 (34.5%)		2051/4421 (46.4%)	
*Male*	1880/2870 (65.5%)		2370/4421 (53.6%)	
**Infection characteristics**				
Mode of acquisition				
*Community-acquired*	1553/2870 (45.9%)		2879/4421 (65.1%)	
*Hospital-acquired*	1317/2870 (54.1%)		1542/4421 (34.9%)	
MRSA/MRGN	228/2870 (7.9%)		681/4421 (15.4%)	
Onset in the ICU	805/2870 (28.0%)		968/4421 (21.9%)	
Polymicrobial infection	171/2870 (6.0%)		461/4421 (10.4%)	

Abbreviations: *ICU, intensive care unit; IQR, inter-quartile range; MRSA, methicillin-resistant S. aureus; 3GCREC, third-generation cephalosporin resistant E. coli.*

### Meteorological characteristics and seasonal variation

**Fig 1:**
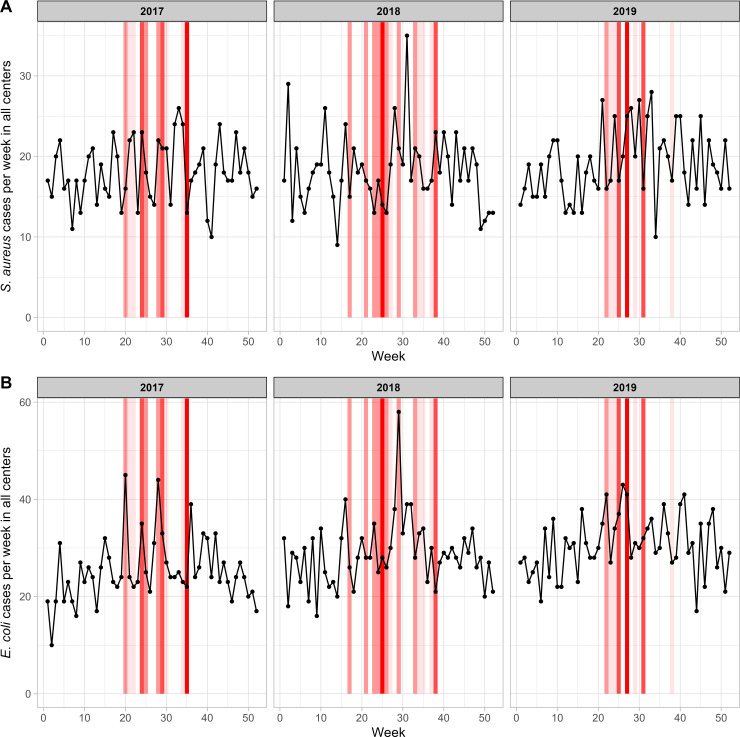
Weekly number of *S. aureus* and *E. coli* bacteremias by year and density of heat days. **A** represents the number of *S. aureus* bacteremia per week aggregated for the years 2017–2019. **B** illustrates the respective numbers for *E. coli* bacteremia. The red beams represent weeks with heat days in all participating centers with an increasing intensity if heat days occurred in more than one center.

Focusing on the meteorological features of the observed time period, 193/6594 days (2.9%) of the period were classified as heat days according to the heat definition used (Fig 1). Heat days occurred mainly between June and August of the three years. The two longest heat waves persisted for twelve days. The mean maximum perceived temperature on heat days was 34.5 °C.

**Fig 2:**
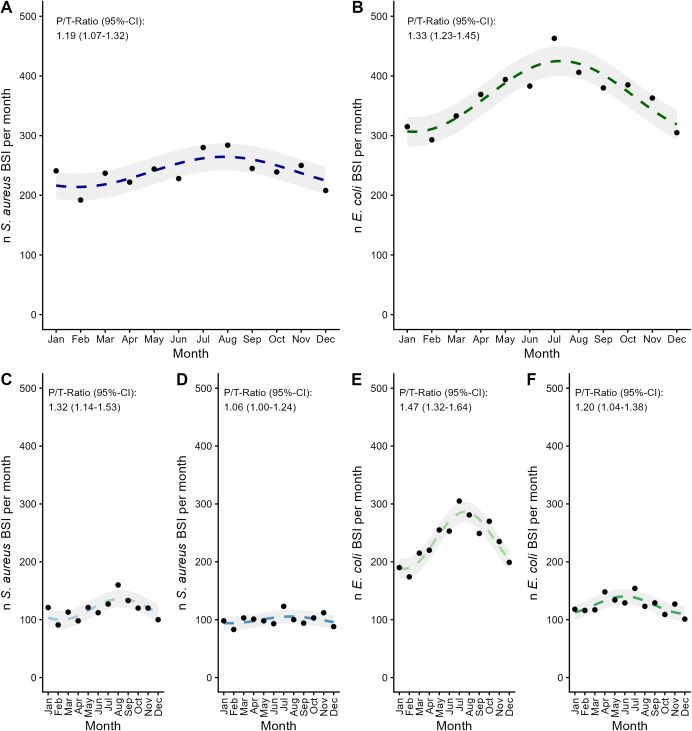
Number of bacteremias aggregated by month for all centers. The fitted curve for each pathogen was modelled by a sinusoidal regression. **A** shows the number of all *S. aureus* bacteremias, while **B** illustrates the number of all *E. coli* bacteremias. The second line of figures displays the case numbers stratified by mode of acquisition and pathogen (**C**: community-acquired cases of *S. aureus* bacteremia; **D** hospital-acquired cases of *S. aureus* bacteremia; **E** community-acquired cases of *E. coli* bacteremia; **F** hospital-acquired cases of *E. coli* bacteremia). The peak-to-trough ratio of the fitted values are shown in the left upper corner of every figure. The following table depicts the regression model estimates for the overall case numbers.

The peak-to-trough ratio for ECB was 1.334 (95% CI: 1.225–1.453, p < 0.001) with maximum of cases in July and minimum of cases in January. SAB showed a slightly lower peak-to-trough ratio of 1.192 (95% CI: 1.074–1.323, p < 0.001), peaking in August and falling to its lowest point in February (Fig 2). Overall, the peak-to-trough ratio was more pronounced for community-acquired bacteremia cases (ECB: 1.468, 95%-CI: 1.318–1.635, p < 0.001; SAB: 1.320 95%-CI: 1.136–1.534, p < 0.001) compared to hospital-acquired cases (ECB: 1.197, 95%-CI: 1.036–1.382, p = 0.014; SAB: 1.059 95%-CI: 1.000–1.243, p = 0.302).

### Negative binominal model of heat waves

**Fig 3:**
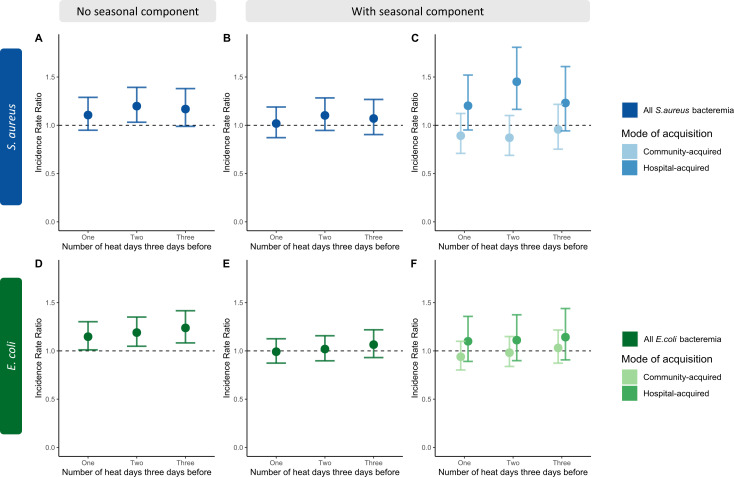
Regression model for heat day effect on *S. aureus* and *E. coli* bacteremias with and without inclusion of seasonal component. **A-C** illustrate the model results of the negative binominal regression model for *S. aureus* bacteremias depending on the number of heat days in the previous three days. **A** does not include a seasonal component in the model, while **B** does include it. **C** shows the stratification of the analysis by mode of acquisition in the model with included seasonal component. **D-F** illustrate the model results of the negative binominal regression model for *E. coli* bacteremias depending on the number of heat days in the previous three days. **D** does not include a seasonal component in the model, while **E** does include it. **F** shows the stratification of the analysis by mode of acquisition in the model with included seasonal component.

In the model without an underlying seasonal trend, we found an increase in the incidence of SAB after previous two or three days of heat (IRR 1.20, p = 0.018; IRR 1.17, p = 0.068; Fig 3A). Concerning ECB, we detected a comparable IRR already after one day of heat exposure (IRR 1.15, p = 0.034; IRR 1.19 after two days, p = 0.007; IRR 1.24 after three days of heat, p = 0.002, Fig 3D). After inclusion of an underlying seasonal trend, the frequency of bacteremia cases remained significantly associated with heat stress days only in the subgroup of patients with hospital-acquired SAB (hospital-acquired SAB: 2d heat: IRR 1.45, p = 0.001, 3d heat: IRR 1.23, p = 0.13; community-acquired SAB: 2d heat: IRR 0.87, p = 0.25, 3d heat: IRR 0.96, p = 0.72, Fig 3B/C). However, with the inclusion of an underlying seasonal trend in the model for ECB a potential impact of heat days was no longer detectable overall (2d heat: IRR 1.02, p = 0.77, 3d heat: IRR 1.07, p = 0.36, Fig 3E) and after stratification by mode of acquisition (hospital-acquired ECB: 2d heat: IRR 1.11, p = 0.33, 3d heat: IRR 1.14, p = 0.26; community-acquired ECB: 2d heat: IRR 0.98, p = 0.82, 3d heat: IRR 1.03, p = 0.72, Fig 3F). In the sensitivity analysis including only summer months and not seasonal modeling component, we found no significant effect of the previous number of heat days on the incidence of SAB or ECB (S1 Fig A/C). Yet, the number of hospital-acquired SAB remained significantly associated with the number of previous heat stress days (2d heat: IRR 1.50, p = 0.001, 3d heat: IRR 1.14, p = 0.43, S4 Fig B)

### Subgroup analyses

Given the observed impact of heat stress days on the incidence of hospital-acquired SAB, we analyzed whether the type of ward at SAB onset may influence this association. We found comparable point estimates after two to three heat days for the subgroups of SAB with onset in the ICU (2d heat: IRR 1.56, p = 0.07, 3d heat: IRR 1.36, p = 0.31) and onset on non-ICU wards (2d heat: IRR 1.43, p = 0.004; 3d heat: IRR 1.20, p = 0.22, S2 Fig), however, as fewer SAB cases with onset in the ICU were included, the confidence intervals were wider and IRRs did not significantly differ after two or three heat days in this subgroup. Furthermore, we found no significant differences in regard to resistance type for *S. aureus* (S3 Fig) and *E. coli* (S4 Fig).

## Discussion

The main findings of our study were we found seasonal variations in the number of SAB and ECBs with peaks in August or July, respectively ECB exhibited a more consistent seasonal pattern with a notable decrease in cases during the colder months. In our setting of temperate climate, heat waves did not have an additional effect on top of seasonal variation on the overall number of SAB or ECB. However, we observed an association between excessive heat days and an increased incidence of hospital-acquired SAB. Finally, from a methodological point of view, our study demonstrates the importance of including a seasonality component in the respective models to prevent misinterpretations when analyzing the impact of heat waves.

Climate change and global warming are major threats for human health. With a global temperature increase of around 2°C by 2050 in even the intermediate future emission scenario presented by the Intergovernmental Panel on Climate Change (IPCC), the frequency of heat waves is highly likely to increase [21]. A growing number of research articles in the past years stressed the probable increase of infectious diseases driven by increasing temperatures, additional to preexisting seasonal changes [2].

Concerning *E. coli*, previous investigations suggested an increase of ECB by temperature and season [4,8,14–17]. In our study, we also found an increase in case numbers during summer months. Nevertheless, after including a general seasonal variable in our model, thereby mimicking underlying seasonal changes, we found no additional association between heat waves and the occurrence of ECBs. A seasonal variable includes season-related differences in behavior and exposition as well as changes of the general temperature and humidity level. Thus, our findings are in favor of an overall seasonal association with the occurrence of ECBs and argue against an additional impact of shorter temperature extremes. Previous studies discussed an increase of *E. coli* in the gut microbiome [22], as well as changes in human behavior during summer months, such as differences in diet and leisure patterns [23]. Further hypothetical mechanisms could be improved growth conditions in non-niche body regions, expression of (additional) virulence genes as well as impairment of innate and adaptive host immune defense mechanisms. Recently, a large population-based study from Israel described no changes in the incidence of ECB (including bacteremia due to multi-drug resistant *E. coli*) depending on temperature or season using the average temperature for all centers [24]. This difference could maybe be explained by the different climate setting with high temperatures throughout the year relative to a temperate setting like in Germany. While population-based data is of high quality in investigating large-scale changes, we would like to highlight the importance of linking local clinical data with high-resolution meteorological data.

For *S. aureus*, seasonal variation in the number of SAB was reported in a Belgian cohort study focusing on relative changes during the year [5]. Yet, other studies yielded conflicting results concerning SAB seasonality aggregating cases by months [4,12,25]. *S. aureus* skin and soft tissue infections (SSTIs) were reported to be more prevalent in summer months, while numbers of respiratory infections were described to be higher in colder months, which may be a consequence of *S. aureus* superinfection in lower respiratory tract infections [26]. With regard to colonization, few studies in healthy individuals found an increased colonization rate of *S. aureus* in skin and respiratory samples during summer, as well as higher MRSA colonization rates in ICU-patients with decreasing latitude, reflecting a possible role of higher temperatures in skin colonization, which is known to constitute a risk factor for subsequent SSTI [12,27,28].

We detected a seasonal variation in SAB cases in our multicenter study using high-resolution meteorological data. Upon adjusting for this seasonality component, hospital-acquired SAB cases were associated with preceding heat days, an association which was not observed for community-acquired SAB cases. In contrast to the intestinal habitat of *E. coli*, the primary niches of *S. aureus* are the anterior nares and the skin. The observed association with heat days could be explained by heat-related effects on the pathogen, like accelerated growth rates or altered expression of genes encoding virulence factors. Heat-sensitive expression of virulence genes such as *hld*, *pms*α, *pms*β, and the *agr*-operon, alpha phenol-soluble modulins (αPSMs) as well as an increment in haemolysis and staphyloxanthin production has been described in *S. aureus* in vitro [29,30]. At the same time heat-related effects on the host could play a role, e.g., pronounced sweating leading to an increased skin permeability, altered host defense mechanisms, or disturbance of the protective skin microbiome of the host. Possible heat-induced changes in the skin microbiome composition could also persist from a community-setting into the hospital context, where healthcare-associated factors such as soaked dressings of indwelling catheters or surgical wounds yielding an additional increased risk of pathogen growth and subsequent infection, which could play a role. As the availability of air conditioning on most non-ICU wards in Germany is limited, direct heat effects are more likely to occur also in the hospital setting compared with other countries and settings where stricter temperature control is the norm. However, two recent small studies found no seasonal variation for catheter-associated bacteremia due to *S. aureus*, yet again with limited generalizability due to a monocentric study design and monthly aggregation of weather data [31,32].

The main strength of the current study is the use of a statistical model that accounts for effect variations by center and, most importantly, seasonal influences, allowing for the first time to differentiate between heat stress days and seasonal effects. Our study was conducted as a multicenter study, which likely increases the validity of our findings. Furthermore, we stratified our analysis by the mode of acquisition as the exposure to heat and other environmental factors is expected to be different for patients in the community compared to an intra-hospital setting, especially with regard to possible portals of entry. Finally, we employed high-resolution meteorological and temporal data integrating local weather data with 3-days resolution, which allowed us to detect short term changes compared to monthly aggregations.

Yet, there are also limitations that need to be taken into account. Due to the observational study design, we are only able to describe an association of hospital-acquired SAB cases with heat days and cannot infer causality. Our study was performed in the temperate climate of Germany, thus, the results cannot be generalized to other regions or climate zones, as influences of temperature and especially heat vary significantly between geographical settings. Although we included three consecutive years in a multicenter design yielding more than 2800 SAB and 4400 ECB episodes, our analysis may still be underpowered to detect a significant impact of heat days. Moreover, we chose to investigate potential consequences of heat phases of 1–3 days with a latency of 3 days. This latency was based on clinical reasoning, as SAB and ECB are considered entities with acute presentations and comparable latencies for other diseases [33-35]. However, longer periods of extreme heat may be required to yield more pronounced effects on host or pathogen physiology. Finally, we used data from close-by weather stations of the respective recruiting hospitals to model the meteorological exposure. Nevertheless, climatic conditions may vary between and even within hospitals. Our strategy to differentiate between ICU (with more efficient climate control/air conditioning units) is a first proxy, however, climate/temperature assessment at the patient/room level will allow for more accurate analyses in future studies.

Further studies are needed to substantiate seasonal trends in SAB and the observed association of hospital-acquired SAB with (previous) heat days. If our findings are confirmed and underlying mechanisms prove to be modifiable, heat protection measures within hospitals may become more important and may evolve into novel infection prevention strategies.

## Conclusion

The current study underlines the relevance of seasonal influences and heat for human health and infections. Large multicenter studies, ideally from different healthcare settings and climate zones are needed to better map a potential impact of heat waves on the incidence of bacteremia and other invasive (vector- or non-vector-borne) infections. Deciphering the underlying mechanisms in host-pathogen interaction may contribute to mitigate climate-associated hazards – especially in countries, which are likely to be affected the hardest by climate change.

## Supporting information

S1 Fig Regression model for heat day effect on *S. aureus* and *E. coli* bacteremias according to mode of acquisition limited to occurrence in summer months. **A-D** illustrate the model results of the negative binominal regression model for *S. aureus* and *E. coli* bacteremias limited to occurrence during summer months without a seasonal component depending on the number of heat days in the previous three days. **A** shows the model for all *S. aureus* bacteremias, while **C** does it for *E. coli* bacteremias. **B** displays the model results for *S. aureus* bacteremia stratified by mode of acquisition, while **D** does it for *E. coli* bacteremias.
(DOCX)

S2 Fig Regression model for heat day effect on hospital-acquired *S. aureus* bacteremias according to onset on ICU or regular ward. Negative binominal model of heat day effect on hospital-acquired *S. aureus* bacteremias according to onset in Intensive Care Units (ICU) or regular wards. **A** illustrates the model results of the negative binominal regression model for *S. aureus* bacteremia depending on the number of heat days in the previous three days and onset of the infection on ICU or regular wards. **B** includes an additional seasonal component in the model.
(DOCX)

S3 Fig Regression model for heat day effect on *S. aureus* bacteremias according to resistance pattern. Negative binominal model of heat day effect on *S. aureus* bacteremias according to resistance pattern. **A** illustrates the model results of the negative binominal regression model for *S. aureus* bacteremia depending on the number of heat days in the previous three days and resistance pattern against methicillin. **B** includes an additional seasonal component in the model.
(DOCX)

S4 Fig Regression model for heat day effect on *E. coli* bacteremias according to resistance pattern. Negative binominal model of heat day effect on *E. coli* bacteremias according to resistance pattern. **A** illustrates the model results of the negative binominal regression model for *E. coli* bacteremia depending on the number of heat days in the previous three days and resistance to third-generation cephalosporins (3GCREC). **B** includes an additional seasonal component in the model.
(DOCX)

S1 Table Number of cases stratified by pathogen, year and center. (DOCX)

S2 Table List of responsible ethics committees. (DOCX)

S1 File Human participants research checklist. (PDF)

**Table:** 

	*S. aureus* bacteremia		*E. coli* bacteremia	
*Explanatory variable*	*β (SE)*	*p-value*	*β (SE)*	*p-value*
Intercept	**218**.**33 (5**.**08)**	**<0**.**001**	**363**.**50 (6**.**01)**	**<0**.**001**
Sine	**−21**.**38 (7**.**19)**	**0**.**02**	**−36**.**04 (8**.**51)**	**0**.**002**
Cosine	−9.27 (7.19)	0.23	**−46**.**11 (8**.**51)**	**<0**.**001**
Model characteristics	R² = 0.54; F-statistic: p-value = **0**.**03**		R² = 0.84; F-statistic: p-value **< 0**.**001**	

Wavelength = 12.
