# Supplementary material for: Impact of seasons and heat waves on the incidence of Staphylococcus aureus and Escherichia coli bacteremia – A prospective multicenter study using biometeorological data
Source: PLoS One. 2026 Jul 14;21(7):e0352186. doi: 10.1371/journal.pone.0352186 (PMC13367701; doi:10.1371/journal.pone.0352186)
Supplement: S1 Fig — A-D illustrate the model results of the negative binominal regression model for S. aureus and E. coli bacteremias limited to occurrence during summer months without a seasonal component depending on the number of heat days in the previous three days. A shows the model for all S. aureus bacteremias, while C does it for E. coli bacteremias. B displays the model results for S. aureus bacteremia stratified by mode of acquisition, while D does it for E. coli bacteremias. (DOCX) [file pone.0352186.s001.docx]

**Supplementary Figure 1: Regression model for heat day effect on *S. aureus* and *E. coli* bacteremias according to mode of acquisition limited to occurrence in summer months**


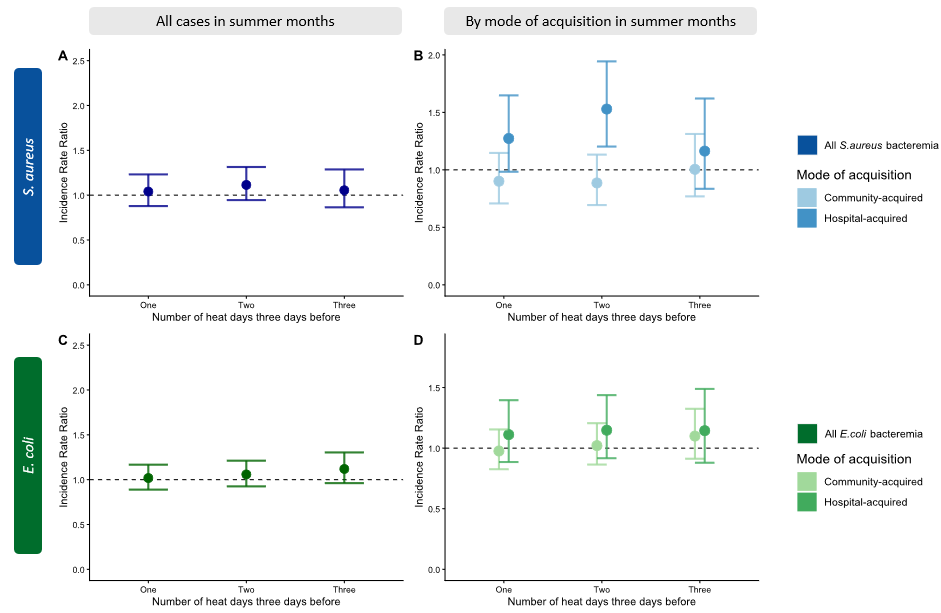


**A-D** illustrate the model results of the negative binominal regression model for *S. aureus* and *E. coli* bacteremias limited to occurrence during summer months without a seasonal component depending on the number of heat days in the previous three days. **A** shows the model for all *S. aureus* bacteremias, while **C** does it for E. coli bacteremias. **B** displays the model results for *S. aureus* bacteremia stratified by mode of acquisition, while **D** does it for *E. coli* bacteremias.
