# Supplementary material for: Impact of seasons and heat waves on the incidence of Staphylococcus aureus and Escherichia coli bacteremia – A prospective multicenter study using biometeorological data
Source: PLoS One. 2026 Jul 14;21(7):e0352186. doi: 10.1371/journal.pone.0352186 (PMC13367701; doi:10.1371/journal.pone.0352186)
Supplement: S2 Fig — Negative binominal model of heat day effect on hospital-acquired S. aureus bacteremias according to onset in Intensive Care Units (ICU) or regular wards. A illustrates the model results of the negative binominal regression model for S. aureus bacteremia depending on the number of heat days in the previous three days and onset of the infection on ICU or regular wards. B includes an additional seasonal component in the model. (DOCX) [file pone.0352186.s002.docx]

**Supplementary Figure 2: Regression model for heat day effect on hospital-acquired *S. aureus* bacteremias according to onset on ICU or regular ward**

**
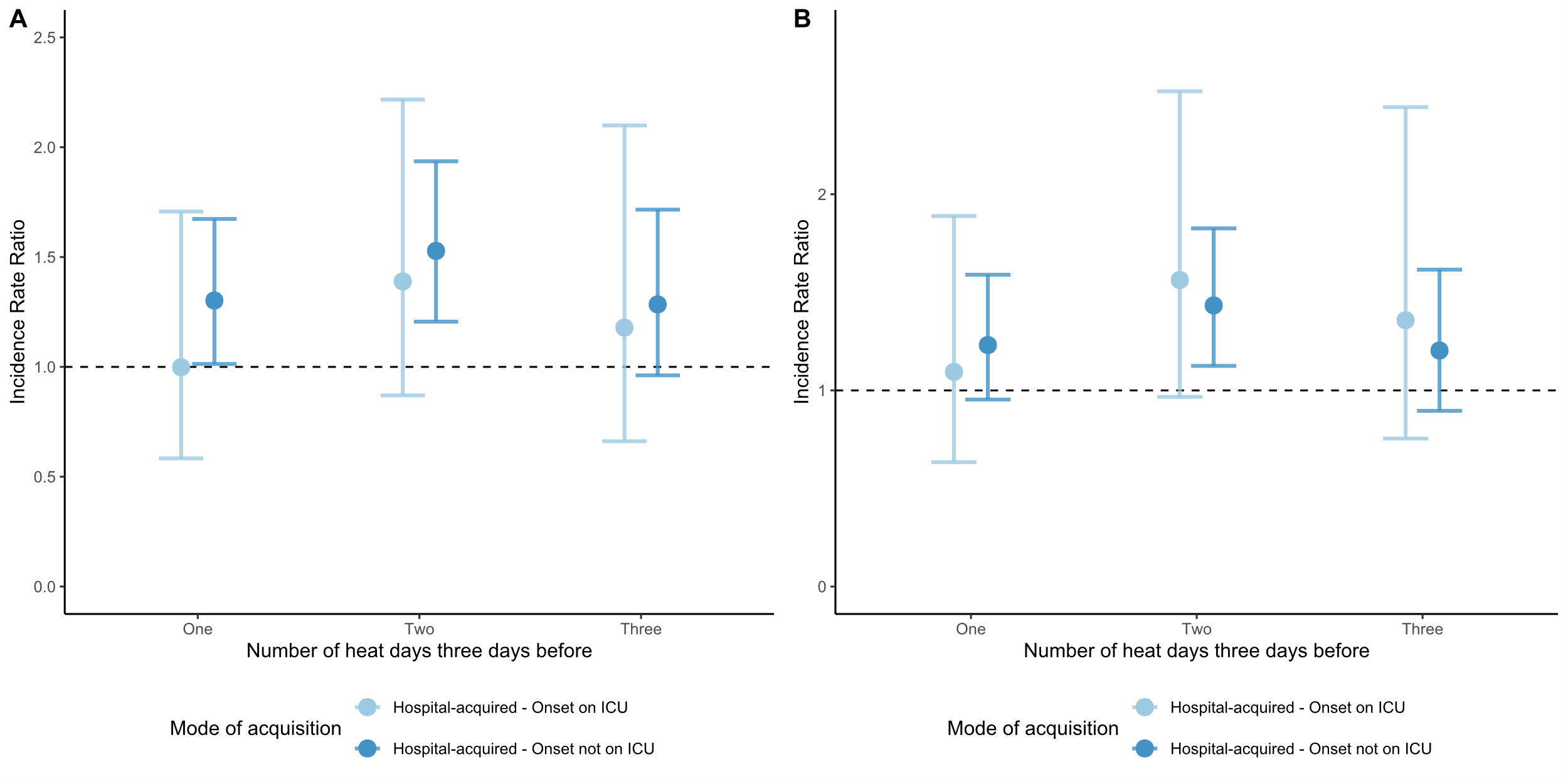
**

|  | ***S. aureus bacteremia - Onset on regular ward*** | | | ***S. aureus bacteremia - Onset on ICU*** | | |
| --- | --- | --- | --- | --- | --- | --- |
| ***Explanatory variable*** | ***β (95%-CI)*** | ***p-value*** | ***Random effects*** | ***β (95%-CI)*** | ***p-value*** | ***Random effects*** |
| Intercept | **0**.**33 (0**.**24-0**.**46)** | **<0**.**001** |  | **0**.**11 (0**.**07-0**.**18)** | **<0**.**001** |  |
| One heat day | 1.23 (0.95-1.59) | 0.111 |  | 1.09 (0.63-1.89) | 0.746 |  |
| Two heat days | **1**.**43 (1**.**13-1**.**83)** | **0**.**004** |  | **1**.**56 (0.97-2**.**53)** | **0**.**068** |  |
| Three heat days | 1.20 (0.90-1.62) | 0.219 |  | 1.36 (0.75-2.44) | 0.307 |  |
| Natural spline, knot 1 | 1.14 (0.97-1.35) | 0.106 |  | 0.77 (0.56-1.05) | 0.095 |  |
| Natural spline, knot 2 | 1.27 (0.92-1.78) | 0.151 |  | 1.10 (0.61-2.02) | 0.746 |  |
| Natural spline, knot 3 | 1.00 (0.87-1.14) | 0.965 |  | 1.17 (0.92-1.47) | 0.198 |  |
| Year 2018 | 1.10 (1.00-1.20) | 0.051 |  | 0.84 (0.71-1.01) | 0.063 |  |
| Year 2019 | 1.08 (0.99-1.19) | 0.099 |  | 1.01 (0.86-1.20) | 0.866 |  |
|  |  |  | σ² = 1.28 |  |  | σ² = 2.38 |
|  |  |  | τ_00_ = 0.13 |  |  | τ_00_ = 0.21 |
|  |  |  | ICC = 0.10 |  |  | ICC = 0.08 |

Negative binominal model of heat day effect on hospital-acquired *S. aureus* bacteremias according to onset in Intensive Care Units (ICU) or regular wards. **A** illustrates the model results of the negative binominal regression model for *S. aureus* bacteremia depending on the number of heat days in the previous three days and onset of the infection on ICU or regular wards. **B** includes an additional seasonal component in the model.
