# Supplementary material for: Impact of seasons and heat waves on the incidence of Staphylococcus aureus and Escherichia coli bacteremia – A prospective multicenter study using biometeorological data
Source: PLoS One. 2026 Jul 14;21(7):e0352186. doi: 10.1371/journal.pone.0352186 (PMC13367701; doi:10.1371/journal.pone.0352186)
Supplement: S3 Fig — Negative binominal model of heat day effect on S. aureus bacteremias according to resistance pattern. A illustrates the model results of the negative binominal regression model for S. aureus bacteremia depending on the number of heat days in the previous three days and resistance pattern against methicillin. B includes an additional seasonal component in the model. (DOCX) [file pone.0352186.s003.docx]

**Supplementary Figure 3: Regression model for heat day effect on *S. aureus* bacteremias according to resistance pattern**

**
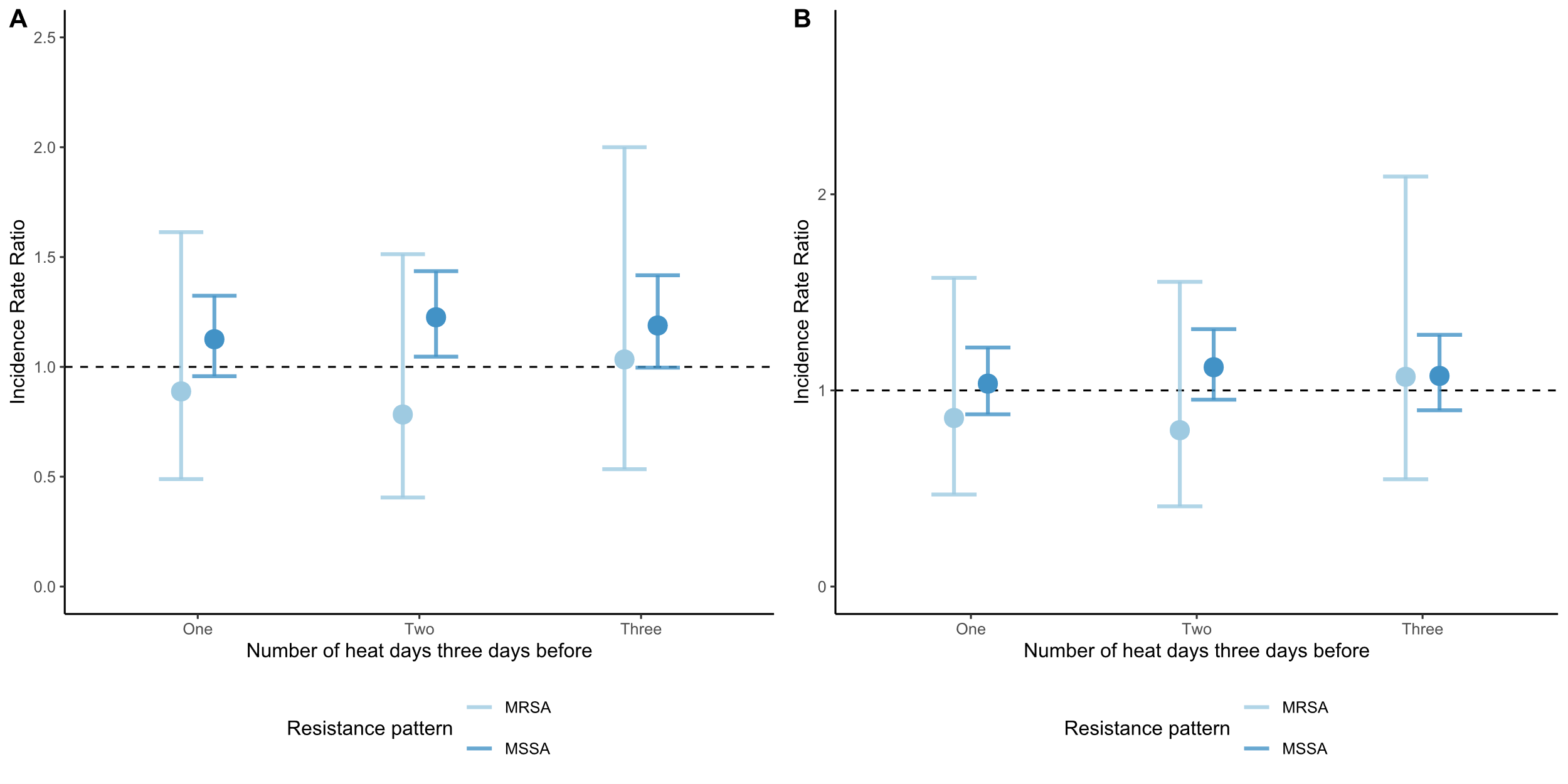
**

|  | ***S. aureus bacteremia - MRSA*** | | | ***S. aureus bacteremia - MSSA*** | | |
| --- | --- | --- | --- | --- | --- | --- |
| ***Explanatory variable*** | ***β (95%-CI)*** | ***p-value*** | ***Random effects*** | ***β (95%-CI)*** | ***p-value*** | ***Random effects*** |
| Intercept | **0.10 (0.05-0.18)** | **<0.001** |  | 0.95 (0.73-1.25) | 0.721 |  |
| One heat day | 0.86 (0.47-1.57) | 0.624 |  | 1.03 (0.88-1.22) | 0.685 |  |
| Two heat days | 0.80 (0.41-1.55) | 0.506 |  | 1.12 (0.95-1.31) | 0.171 |  |
| Three heat days | 1.07 (0.55-2.09) | 0.845 |  | 1.07 (0.90-1.28) | 0.432 |  |
| Natural spline, knot 1 | 1.09 (0.79-1.51) | 0.599 |  | **1.35 (1.23-1.49)** | **<0.001** |  |
| Natural spline, knot 2 | 1.31 (0.68-2.53) | 0.421 |  | 1.03 (0.85-1.26) | 0.755 |  |
| Natural spline, knot 3 | **0.66 (0.50-0.88)** | **0.005** |  | 1.03 (0.95-1.12) | 0.449 |  |
| Year 2018 | **0.66 (0.55-0.79)** | **<0.001** |  | **1.09 (1.03-1.15)** | **0.004** |  |
| Year 2019 | **0.66 (0.55-0.80)** | **<0.001** |  | **1.13 (1.07-1.20)** | **<0.001** |  |
|  |  |  | σ² = 2.55 |  |  | σ² = 0.66 |
|  |  |  | τ_00_ = 0.47 |  |  | τ_00_ = 0.10 |
|  |  |  | ICC = 0.16 |  |  | ICC = 0.14 |

Negative binominal model of heat day effect on *S. aureus* bacteremias according to resistance pattern. **A** illustrates the model results of the negative binominal regression model for *S. aureus* bacteremia depending on the number of heat days in the previous three days and resistance pattern against methicillin. **B** includes an additional seasonal component in the model.
