# Supplementary material for: Impact of seasons and heat waves on the incidence of Staphylococcus aureus and Escherichia coli bacteremia – A prospective multicenter study using biometeorological data
Source: PLoS One. 2026 Jul 14;21(7):e0352186. doi: 10.1371/journal.pone.0352186 (PMC13367701; doi:10.1371/journal.pone.0352186)
Supplement: S4 Fig — Negative binominal model of heat day effect on E. coli bacteremias according to resistance pattern. A illustrates the model results of the negative binominal regression model for E. coli bacteremia depending on the number of heat days in the previous three days and resistance to third-generation cephalosporins (3GCREC). B includes an additional seasonal component in the model. (DOCX) [file pone.0352186.s004.docx]

**Supplementary Figure 4: Regression model for heat day effect on *E. coli* bacteremias according to resistance pattern**

**
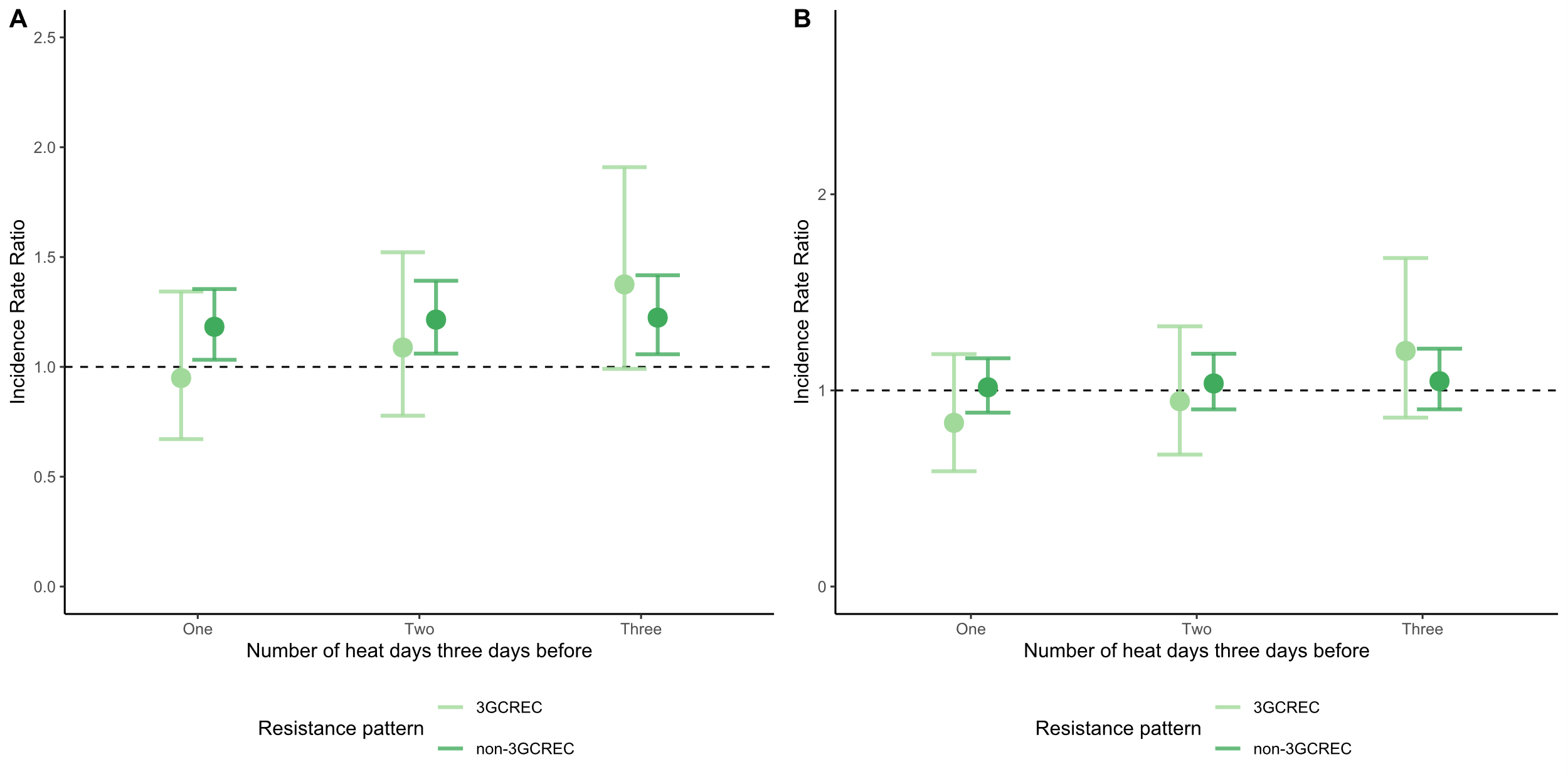
**

|  | ***E. coli bacteremia – 3GCREC*** | | | ***E. coli bacteremia – non-3GCREC*** | | |
| --- | --- | --- | --- | --- | --- | --- |
| ***Explanatory variable*** | ***β (95%-CI)*** | ***p-value*** | ***Random effects*** | ***β (95%-CI)*** | ***p-value*** | ***Random effects*** |
| Intercept | **0.17 (0.12-0.23)** | **<0.001** |  | 1.07 (0.78-1.48) | <0.673 |  |
| One heat day | 0.83 (0.59-1.19) | 0.312 |  | 1.02 (0.89-1.16) | 0.820 |  |
| Two heat days | 0.94 (0.67-1.33) | 0.744 |  | 1.04 (0.90-1.19) | 0.617 |  |
| Three heat days | 1.20 (0.86-1.68) | 0.280 |  | 1.05 (0.90-1.21) | 0.541 |  |
| Natural spline, knot 1 | **1.43 (1.17-1.74)** | **<0.001** |  | **1.46 (1.34-1.58)** | **<0.001** |  |
| Natural spline, knot 2 | **1.80 (1.19-2.73)** | **0.006** |  | **1.66 (1.39-1.98)** | **<0.001** |  |
| Natural spline, knot 3 | 1.15 (0.98-1.35) | 0.077 |  | **0.89 (0.83-0.96)** | **<0.001** |  |
| Year 2018 | **1.27 (1.13-1.43)** | **<0.001** |  | **1.13 (1.08-1.19)** | **<0.001** |  |
| Year 2019 | **1.44 (1.28-1.62)** | **<0.001** |  | **1.19 (1.13-1.25)** | **<0.001** |  |
|  |  |  | σ² = 1.57 |  |  | σ² = 0.53 |
|  |  |  | τ_00_ = 0.10 |  |  | τ_00_ = 0.15 |
|  |  |  | ICC = 0.06 |  |  | ICC = 0.22 |

Negative binominal model of heat day effect on *E. coli* bacteremias according to resistance pattern. **A** illustrates the model results of the negative binominal regression model for *E. coli* bacteremia depending on the number of heat days in the previous three days and resistance to third-generation cephalosporins (3GCREC). **B** includes an additional seasonal component in the model.
