# Supplementary material for: Impact of seasons and heat waves on the incidence of Staphylococcus aureus and Escherichia coli bacteremia – A prospective multicenter study using biometeorological data
Source: PLoS One. 2026 Jul 14;21(7):e0352186. doi: 10.1371/journal.pone.0352186 (PMC13367701; doi:10.1371/journal.pone.0352186)
Supplement: S1 Table — (DOCX) [file pone.0352186.s005.docx]

**Supplementary table 1: Number of cases stratified by pathogen, year and center**

|  | ***S. aureus*** | | | ***E. coli*** | | |
| --- | --- | --- | --- | --- | --- | --- |
|  | *2017* | *2018* | *2019* | *2017* | *2018* | *2019* |
| **Center A** | 165 | 173 | 191 | 263 | 272 | 310 |
| **Center B** | 277 | 249 | 247 | 437 | 469 | 529 |
| **Center C** | 166 | 168 | 184 | 213 | 207 | 212 |
| **Center D** | 129 | 126 | 133 | 146 | 166 | 175 |
| **Center E** | 126 | 147 | 136 | 180 | 177 | 192 |
| **Center F** | 80 | 72 | 101 | 123 | 179 | 171 |
| **Total** | **943** | **935** | **992** | **1362** | **1470** | **1589** |
