# Supplementary material for: Impact of seasons and heat waves on the incidence of Staphylococcus aureus and Escherichia coli bacteremia – A prospective multicenter study using biometeorological data
Source: PLoS One. 2026 Jul 14;21(7):e0352186. doi: 10.1371/journal.pone.0352186 (PMC13367701; doi:10.1371/journal.pone.0352186)
Supplement: S2 Table — (DOCX) [file pone.0352186.s006.docx]

**Supplementary Table 2: List of responsible Ethics committees**

Ethics committee of the Albert-Ludwigs-University Freiburg

Ethics committee of the Charité

Ethics committee of the Justus-Liebig-University Gießen

Ethics committee of the University of Lübeck

Ethics committee of the University of Cologne

Ethics committee of the University of Tübingen)
